# Hypofractionated radiotherapy for primary or secondary oligometastatic lung cancer using Tomotherapy

**DOI:** 10.1186/1748-717X-7-222

**Published:** 2012-12-27

**Authors:** Heng-Jui Chang, Hui-Ling Ko, Cheng-Yen Lee, Ren-Hong Wu, Yu-Wung Yeh, Jiunn-Song Jiang, Shang-Jyh Kao, Kwan-Hwa Chi

**Affiliations:** 1 Department of Radiation Therapy and Oncology, Shin-Kong Wu Ho-Su Memorial Hospital, 95, Wen-Chang Road, Shih-Lin, Taipei City, Taiwan; 2 Department of Chest Medicine, Shin-Kong Wu Ho-Su Memorial Hospital, Taipei, Taiwan; 3 Faculty of Medicine, College of Medicine, National Yang-Ming University, Taipei, Taiwan

**Keywords:** Tomotherapy, Hypofractionation, Lung cancer, Oligometastasis, Extra-pulmonary disease

## Abstract

**Background:**

To retrospectively review the outcome of patients with primary or secondary oligometastatic lung cancer, treated with hypofractionated Tomotherapy.

**Methods:**

Between April 2007 and June 2011, a total of 33 patients with oligometastatic intrapulmonary lesions underwent hypofractionated radiotherapy by Tomotherapy along with appropriate systemic therapy. There were 24 primary, and 9 secondary lung cancer cases. The radiation doses ranged from 4.5 to 7.0 Gy per fraction, multiplied by 8–16 fractions. The median dose per fraction was 4.5 Gy (range, 4.5-7.0 Gy), and the median total dose was 49.5 Gy (range, 45–72 Gy). The median estimated biological effective dose at 10 Gy (BED_10_) was 71.8 Gy (range, 65.3–119.0 Gy), and that at 3 Gy (BED_3_) was 123.8 Gy (range, 112.5–233.3 Gy). The mean lung dose (MLD) was constrained mainly under 1200 cGy. The median gross tumor volume (GTV) was 27.9 cm^3^ (range: 2.5–178.1 cm^3^).

**Results:**

The median follow-up period was 25.8 months (range, 3.0–60.7 months). The median overall survival (OS) time was 32.1 months for the 24 primary lung cancer patients, and >40 months for the 9 metastatic lung patients. The median survival time of the patients with extra-pulmonary disease (EPD) was 11.2 months versus >50 months (not reached) in the patients without EPD (p < 0.001). Those patients with smaller GTV (≦27.9 cm^3^) had a better survival than those with larger GTV (>27.9 cm^3^): >40 months versus 12.85 months (p = 0.047). The patients with ≦2 lesions had a median survival >40 months, whereas those with ≧3 lesions had 26 months (p = 0.065). The 2-year local control (LC) rate was 94.7%. Only 2 patients (6.1%) developed ≧grade 3 radiation pneumonitis.

**Conclusion:**

Using Tomotherapy in hypofractionation may be effective for selected primary or secondary lung oligometastatic diseases, without causing significant toxicities. Pulmonary oligometastasis patients without EPD had better survival outcomes than those with EPD. Moreover, GTV is more significant than lesion number in predicting survival.

## Introduction

Many cancer patients succumb to either primary or secondary lung cancer. Primary lung cancer is the leading cause of cancer-related mortality all over the world. It results in 20% of all cancer deaths in Taiwan, and approximately 25–30% in the United States [1]. Secondary lung cancer is observed in 30-60% of all cancer patients, although prevalence varies on the basis of different primary cancer. Approximately 75–80% of primary lung cancer patients have locally advanced stage III or IV disease at diagnosis, and late diagnosis has been an obstacle to improving survival in the past several decades. However, primary lung cancer outcome has been greatly improved in recent years owing to the introduction of targeted and chemotherapy. Likewise, improved distant control and overall survival of secondary lung cancer has been perceived.

Given the continued improvement in systemic therapy for lung cancer treatment, the role of local therapy may be more important because oligometastasis or oligorecurrence states are more frequent in the era of good but not curative systemic therapy [2].

The paradigm of “oligometastasis” has long been established in lung metastases from sarcoma and liver metastases from colorectal cancer by Hellman and Weichselbaum [3,4]. An intermediate state between purely localized lesions and widespread systemic disease does exist in many types of cancers. Before the cancer cells acquire the ability to spread throughout the whole body, local resection or ablative therapy with curative intent may reduce the tumor burden and prolong the lives of patients. By definition, the oligometastasis should be confined to a small number of tumors in a limited number of organs. The detectable tumor sites should be removed by surgery or radiation therapy.

Stereotactic radiation therapy (SRT) is an external beam radiation procedure that has been widely used since the 1990’s. It was initially used in small intracranial or spinal tumors in a single fraction. Later, the technique was applied to tumors outside the brain or spine, using a stereotactic body frame, with 6–22 Gy/per fraction, multiplied by 3–5 fractions [5]. This technique is called stereotactic body radiation therapy (SBRT). Previous studies evaluating SBRT for lung cancer mainly involved T1–T2N0, medically inoperable patients with a 2-year local control rate of approximately 90–95%, and varied OS rate (3-years: 40–70%) [6-9]. Regarding oligometastasis treated by SBRT, the weighted 2-year LC rate of: 77.9% (67–96%), and weighted 2-year OS rate of 53.7% (33–89%) were presented in a systemic review [10].

The concept of SBRT has also been transformed into hypofractionated radiotherapy (HRT) with a conventional immobilization cast and a treatment course of usually more than 8 fractions by the emergence of image-guided techniques. The reliability of treating lung tumors by HRT with a conventional immobilization cast can be much improved by Tomotherapy. There are 2 advantages of Tomotherapy: (1) image-guided technique: megavoltage computed tomography (MVCT) before each treatment may be easily applied, and precise daily tumor targeting may avoid the stringent hypofractionated schedule; (2) MVCT is a slow CT scan that may more accurately captured cephalad and caudal margins during respiratory movement and is suitable for treatment planning.

The efficacy and feasibility of HRT by Tomotherapy with a conventional immobilization cast has not yet been widely reported. In this study, we retrospectively reviewed our experience of treating primary or secondary lung cancer with Tomotherapy in hypofractionation. We assess the LC rate, biological effective dose, overall survival and toxicities.

## Materials and methods

### Patient characteristics

We retrospectively evaluated 33 patients who underwent HRT with Tomotherapy (TomoTherapy Incorporated, Madison, WI) for primary and secondary lung tumors with curative intent at Shin Kong Hospital between April 2007 and June 2011. Secondary lung cancer is cancer that originates in somewhere else in the body and spreads to the lungs. Operability was discussed by a multidisciplinary board before treatment. All patients have signed the consent before the treatment. Bone scans and FDG-PET scans were performed in selected patients. Our department policies of using PET/CT as complementary tool on primary or secondary lung cancer before radiotherapy are basically based on the following criteria: (1) to determine the resectability (2) to determine the state of oligometastasis (3) to guide for radiotherapy field. Fewer than 5 targets inside the thoracic region were required. The size of each primary tumor was required to be <5 cm. Lung-to-lung metastasis or mediastinal lymph node lesions were allowed, but overall, there should be less than 5 individual gross tumor volumes (GTVs).

### MVCT simulation

All the patients were immobilized using conventional thermoplastic frames with a diaphragm compressor to control the amplitude of respiration to less than 15 mm, which was investigated under fluoroscopy. MVCT was performed for treatment planning in slices of 3-mm thickness, and the images were then transferred to the Tomotherapy planning station, HiArt version 4.04. All MVCT scans were fused with conventional CT or PET/CT scans for contouring.

### Delineation of target volumes

The planning target volume (PTV) was 3 mm added to the clinical target volume (CTV), which covered the GTV plus an expansion of 5 mm from MVCT. The normal organs at risk (OAR) for potential radiation injury must be contoured. For lung tumors, the OAR may include (1) the spinal cord, (2) esophagus, (3) heart, and (4) lung. At least 93% of the prescription dose was required to cover 99% of the PTV. The maximum dose could not exceed 125% of the prescribed dose while fulfilling dose constraints for the lung (each mean lung dose [MLD] < 15 Gy, V_5Gy_ < 42%), spinal cord (Dmax < 30 Gy), and heart (mean < 26 Gy). To calculate the MLD, a contour comprising the volume of each lung minus the GTV was constructed.

### Dose-volume analysis of treatment plans

The prescribed doses for primary lung cancers were between 450 and 500 cGy per fraction for 8–16 fractions for lung parenchymal masses and 300 cGy per fraction with 8–16 fractions for mediastinal lymph nodes. The doses for metastatic lung cancers ranged from 450 to 700 cGy per fraction for 8–16 fractions. MLD was controlled mainly under 1200 cGy according to the lung cancer radiotherapy protocol in our department. The Tomotherapy treatment plan was a pitch of 0.287, a width jaw setting of 2.5 cm, and a modulation factor of 3.0. All the plans were assessed and doubly checked by 2 individual medical physicists. Once the optimization was completed, the radiation oncologist reviewed the isodose distributions for final approval of the treatment plans. According to general recommendations, the biological effective dose (BED) calculations are at 3 Gy (BED_3_) for late-responding tissue and radiation pneumonitis (RP), and at 10 Gy (BED_10_) for lung tumors. We used the formula from Dale et al. [11], in which the adopted *K* value of 0.9 Gy day^−1^ represents the BED required each day (after *T*_delay_ has been passed) to offset repopulation, and *T*_delay_ has a working value of 28 days.

### Systemic therapy

Most patients underwent systemic therapy including chemotherapy/tyrosine kinase inhibitor (TKI) before and after radiotherapy. The thoracic irradiation of the patients who developed extrapulmonary disease (EPD) within 3 months after the treatment was considered as ineffective systemic therapy, and the treatment results were analyzed as EPD-positive.

### Toxicity and tumor control

All the patients were evaluated at least once a week during the radiotherapy (RT) period for acute toxicity. Follow-up lung CT scans were carried out at least 1.5 months after the completion of radiotherapy and at 3-month intervals thereafter. The RP grade was evaluated according to the Acute Radiation Morbidity Scoring Criteria and The Radiation Therapy Oncology Group (RTOG)/European Organisation for Research and Treatment of Cancer (EORTC) Late Radiation Morbidity Scoring Schema. The tumor response was assessed according to the Response Evaluation Criteria In Solid Tumors (RECIST) criteria. The recurrence patterns were classified as “in-field” if >95% of the volume was within the 95% isodose, “marginal” if 20–95% of the volume was within the 95% isodose, or “outside” if <20% of the volume was inside the 95% isodose [12].

### Statistical methods

Kaplan-Meier estimates of survival curves were computed for median OS. Comparisons of median OS rates were performed using the log-rank test. The Pearson correlation was also calculated to reflect the degree of the linear relationship between 2 different variables. Owing to the small number of subjects, a multivariable analysis was not conducted. The SPSS (version 13) software was used for all the data analyses.

## Results

### Patient characteristics

As shown in Table 1, 24 male and 9 female patients, with a median age of 68 years (range: 31–82 years), were evaluated. The median follow-up period was 25.8 months (range, 3.0–60.7 months). Twenty-four patients had primary lung cancer (72.7%), and 1 patient had recurrent mesothelioma; 8 secondary lung cancer cases included 3 from the colorectum, 1 from the esophagus, 1 from the stomach, 1 from the liver, 1 from the head and neck, and 1 from sarcoma. The 24 primary lung cancer patients were all stage IV cancer. Eighteen cases (75.0%) were adenocarcinoma; 3 (12.5%), squamous cell carcinoma; and 3 (12.5%), unspecified non-small cell lung cancer (NSCLC). The data of tumor volume, healthy lung volume, and max spinal cord dose were illustrated in Table 2. The median GTV was 27.9 cm^3^ (range: 2.5–178.1 cm^3^). The median healthy lung volume was 2309.0 cm^3^ (range: 1235.5–4221.3 cm^3^). The median max spinal cord dose was 14.1 Gy (range: 2.0-37.4 Gy), and only 2 patients exceeded 30 Gy.

**Table 1:** Patients characteristics

**Variable**	**Distribution**	**Numbers**
Sex	Male	24
	Female	9
Age (years)	Range	31-82
	Median	68
Performance Status	0	20
	1	10
	2	3
Primary tumor site	Lung	24
	Mesothelioma	1
	Head and neck	1
	Colorectum	3
	Esophagus	1
	Stomach	1
	Liver	1
	Sarcoma	1
Primary lung cancer	Stage IV	24
Extrapulmonary disease	No	18
	Yes	15
No of total RT targets	1	20
	2	6
	3	3
	4	1
	5	3
Concurrent systemic therapy	No	10
	Yes	23

**Table 2:** Data of tumor volume, healthy lung volume, and Dmax of spinal cord

**Case**	**GTV (cm**^**3**^**)**	**R’t lung-GTV (cm**^**3**^**)**	**L’t lung-GTV (cm**^**3**^**)**	**Whole lung-GTV (cm**^**3**^**)**	**Max spinal cord dose (Gy)**
1	146.9	1227.1	771.7	1998.8	21.0
2	11.3	1638.8	1640.0	3278.8	16.0
3	50.7	1308.0	1111.1	2419.1	28.7
4	52.0	2183.1	2038.2	4221.3	18.3
5	65.5	682.9	632.1	1315.0	9.6
6	12.8	1323.6	985.4	2309.0	18.9
7	4.1	776.9	458.6	1235.5	14.2
8	19.8	936.2	892.9	1829.1	12.1
9	28.9	1577.6	1164.8	2742.4	37.4
10	70.6	2187.3	1990.3	4177.6	19.6
11	45.4	1720.7	1591.6	3312.3	2.0
12	69.2	2163.3	1407.4	3570.7	17.6
13	6.8	1012.7	536.7	1549.4	11.6
14	27.9	955.1	955.2	1910.3	19.7
15	18.9	1155.7	1443.7	2599.4	6.8
16	20.6	1042.4	1180.7	2223.1	13.8
17	35.1	1333.5	1102.1	2435.6	20.5
18	36.9	1213.7	938.0	2151.7	17.0
19	39.2	1742.5	1861.8	3604.3	14.1
20	12.5	1346.6	1231.2	2577.8	3.8
21	13.2	1725.0	1245.3	2970.3	11.5
22	178.1	2022.6	987.2	3009.8	36.6
23	27.9	1408.5	853.5	2262.0	10.9
24	3.9	1057.3	605.3	1662.6	7.4
25	6.4	681.7	657.6	1339.3	10.4
26	47.9	1266.1	774.7	2040.8	13.0
27	47.7	1741.8	1341.4	3083.2	18.3
28	2.5	833.9	1102.0	1935.9	4.4
29	30.0	1267.3	816.9	2084.2	8.5
30	13.2	1052.1	617.5	1669.6	23.1
31	44.4	2250.2	1849.8	4100.0	9.1
32	27.0	1978.7	1582.0	3560.7	26.1
33	20.7	1153.0	980.5	2133.5	11.6

### Chemotherapy/target therapy

Among the 24 lung cancer patients, all underwent systemic treatment before and after radiotherapy.

Eighteen patients (75%) underwent concurrent systemic therapy during radiotherapy. The mainstay regimen of all 24 patients included: TKI (either Iressa or Tarceva) in 13 patients, Navelbine in 7 patients, Alimta in 2 patients, and Cyclophosphamide (Endoxan) + Uracil-Tegafur (UFUR) in 2 patients. No patients received gemcitabine (Gemzar) based regimen during radiotherapy course. All the adenocarcinoma patients received TKI in the course of their long-term treatment, which might be concurrently with, before, or after radiotherapy. The 9 other patients had secondary lung cancers, of whom 3 underwent RT alone (33.3%), 2 received thalidomide, 3 received capecitabine (Xeloda) based regimen, and 1 received Uracil-Tegafur (UFUR) alone. All the patients with primary lung cancer and 3 patients with metastatic colorectal cancer continued their systemic therapy programs.

### Total radiation doses and treatment duration

The median fraction size was 4.5 Gy (range, 4.5–7.0 Gy/fx), and the median total dose for lung oligometastasis was 49.5 Gy (range, 45–72 Gy). The median overall treatment time (*T*) was 14.5 days (range, 10–27 days). The median estimated BED_10_ was 71.8 Gy (range, 65.3–119.0 Gy), and median estimated BED_3_ was 123.8 Gy (range, 112.5–233.3 Gy).

### Treatment outcome

**Figure 1:**
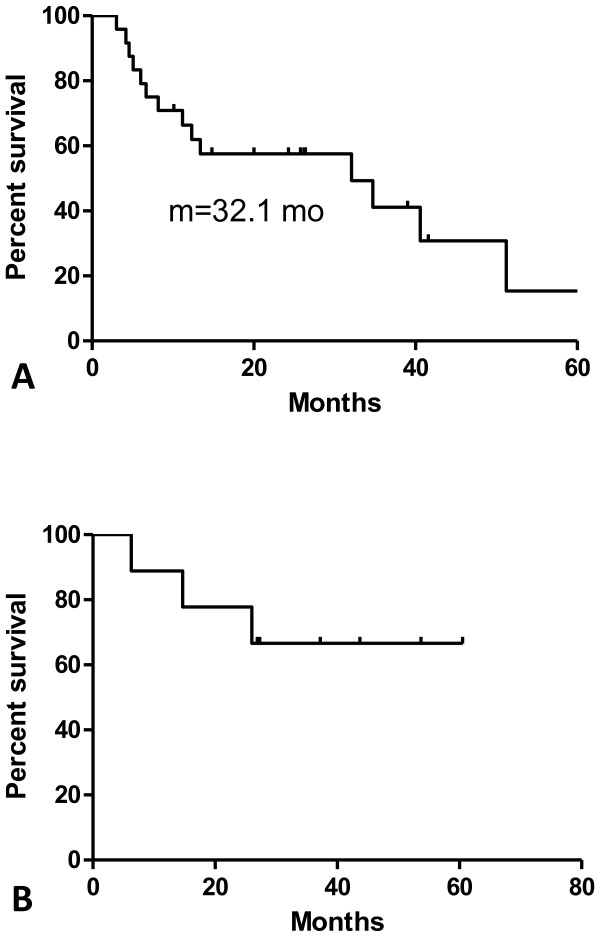
**(A) Overall survival in oligometastatic primary lung cancer patients (n = 24).** The median survival was 32.1 months. (**B**) Overall survival in secondary lung cancer patients (n = 9). Median survival was not reached.

**Figure 2:**
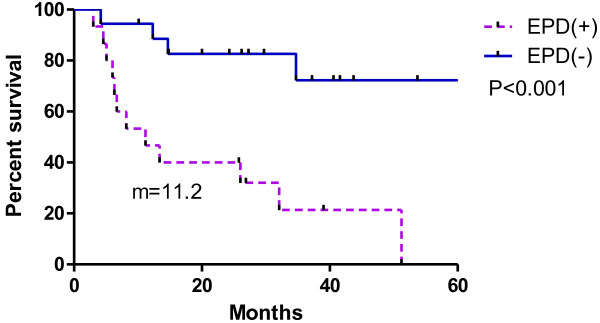
**Relationship between EPD status and overall survival rate.** The EPD (−) group did not achieve median overall survival, while the overall survival in the EPD (+) group was only 11.2 months (p < 0.001).

**Figure 3:**
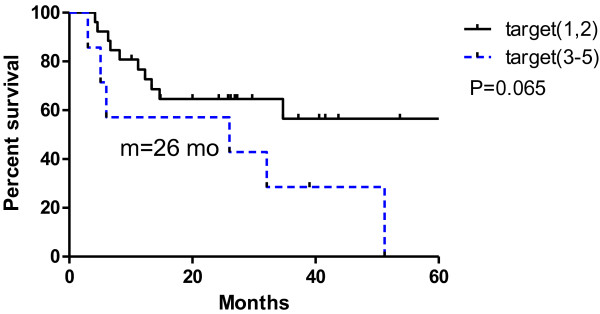
**Overall survival difference of 2 and 3 total oligometastatic lesions in all 33 patients.** Median survival were undefined and 26 months separately (p = 0.065).

**Figure 4:**
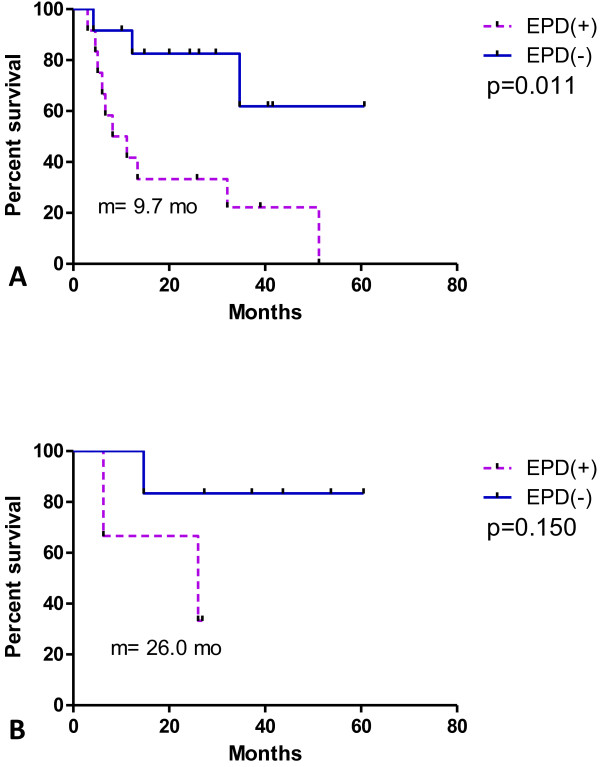
**(A) EPD status in primary lung cancer also leads survival difference.** EPD(+) vs EPD(−): 9.7 months vs not reached (p = 0.011). (**B**) EPD status in secondary lung cancer. EPD(+) vs EPD(−): 26 months vs not reached (p = 0.150).

As illustrated in Figure 1A and Table 3, the median survival of the patients with primary lung cancers (n = 24) was 32.1 months. The overall survival rates at 1, 2, 3, and 4 years were 65.2%, 55.9%, 39.9%, and 39.9%, respectively. As shown in Figure 1B and Table 4, the secondary lung cancer patients (n = 9) had a median survival > 40 months (not reached), and the overall survival rates at 1, 2, 3, and 4 years were 88.9%, 77.8%, 66.7%, and 66.7%, respectively. As shown in Figure 2, the patients with EPD (n = 15) had a median survival of 11.2 months, whereas those without EPD (n = 18) had a longer median survival (>50 months, not reached; p < 0.001). We also analyzed the relationship between the numbers of intrathoracic metastatic lesions of ≤2 and ≥3 in Figure 3. There was no significant difference in survival between the 2 groups: >40 months (not reached) versus 26 months (p = 0.065). As shown in Figure 4A and B, the influence of EPD status on overall survival was significant in the primary lung cancer cases but not in the secondary lung cases. In the primary lung cancer group, the survival of the EPD-negative group versus the EPD-positive group was >50 months (not reached) versus 9.7 months, respectively (p = 0.011). At the end point of the follow-up period, 33 patients with 65 intrapulmonary lesions were investigated, and only 1 lesion had in-field local recurrence. The 2-year LC rate was 94.7%. Tomotherapy without systemic treatment may be curative for secondary lung metastatic lesions without EPD. One sarcoma patient was still alive 60.5 months after RT, 1 rectal cancer patient was still alive 53.7 months after RT, and 1 colon cancer patient was still alive 43.7 months after RT.

**Table 3:** Characteristics of oligometastatic primary lung cancer (n = 24) receiving Tomotherapy

**Case**	**Sex**	**Age**	**Stage**	**Pathological diagnosis**	**Dose (cGy x fx)**	**Intrathoracic Targets**	**Major Systemic Therapy**	**Status**	**Survival time (mo)**
1	F	53	T4N2M1,IV	NSCLC	450 × 10	5	TKI	D	51.2
2	M	58	M1, IV	AD	450 × 10	2	TKI	D	40.6
3	M	31	M1, IV	AD	450 × 10	3	CT (Alimta)	A	39.0
4	M	59	M1, IV	AD	450 × 11	3	CT (Navelbine)	D	6.0
5	M	43	M1, IV	AD	450 × 10	1	TKI	D	13.4
6	M	79	T1NxM1, IV	AD	500 × 9	2	CT(Endoxan + UFUR)	D	8.2
7	F	49	T1N0M1, IV	AD	500 × 9	1	CT (Alimta)	A	29.7
8	F	80	M1, IV	AD	450 × 10	1	TKI	D	4.6
9	M	69	M1, IV	AD	450 × 12	5	CT (Navelbine)	D	5.1
10	M	79	T2N0M1,IV	NSCLC	450 × 16	1	CT (Navelbine)	D	6.7
11	M	64	T2N0M1, IV	AD	550 × 10	1	TKI	D	11.2
12	M	71	rT4, IV	SqCC	500 × 12	1	CT (Navelbine)	D	12.3
13	F	74	T4N2M1,IV	AD	450 × 10	1	TKI	A	26.2
14	M	73	rT1a,IV	AD	450 × 10	1	TKI	A	24.3
15	M	67	rT3,IV	NSCLC	500 × 10	1	CT(Endoxan + UFUR)	A	14.8
16	F	70	M1,IV	AD	500 × 9	2	TKI	A	20.0
17	F	45	M1,IV	AD	550 × 10	1	TKI	D	34.7
18	M	50	T2aN0M1,IV	AD	500 × 10	1	TKI	A	25.8
19	M	78	M1,IV	SqCC	450 × 10	1	CT (Navelbine)	D	4.2
20	M	47	rN1,IV	AD	450 × 10	1	TKI	A	41.6
21	M	68	T1N2M1,IV	AD	450 × 10	5	TKI	D	32.1
22	M	79	M1,IV	AD	450 × 10	3	CT (Navelbine)	D	3.0
23	F	79	rT3, IV	SqCC	450 × 12	2	CT (Navelbine)	A	60.7
24	F	58	M1b,IV	AD	450 × 10	1	TKI	A	10.1

*M* = male; *F* = female; *AD* = adenocarcinoma; *SqCC* = squamous cell carcinoma; *NSCLC* = non-small cell lung cancer; *fx* = fractions; *A* = alive; *D* = dead; *TKI* = tyrosine kinase inhibitor (Erlotinib or Gefitinib); *CT* = chemotherapy.

**Table 4:** Characteristics of oligometastatic secondary cancer (n = 9) receiving Tomotherapy

**Case**	**Sex**	**Age**	**Stage**	**Pathological diagnosis**	**Dose (cGy x fx)**	**Intrathoracic Targets**	**Major systemic therapy**	**Status**	**Survival time (mo)**
1	M	68	IVB	HCC	500 × 8 + 450 × 3	1	Thalidomide	A	26.9
2	M	51	IV	Sarcoma	700 × 10	1	Thalidomide	A	60.5
3	M	71	IVC	H&N SqCC	500 × 10	1	-	D	6.3
4	M	61	IV	Esophagus SqCC	600 × 8	1	-	D	14.7
5	M	70	-	Mesothelioma	500 × 10	1	-	A	27.3
6	M	77	IV	Colon AD	550 × 10	2	Xeloda	A	43.7
7	M	82	IV	Rectum AD	450 × 13	1	Xeloda	A	53.7
8	M	63	IV	Stomach AD	450 × 12	2	Xeloda	A	37.2
9	F	77	IV	Rectum AD	450 × 9	4	UFUR	D	26.0

*M* = male; *F* = female; *AD* = adenocarcinoma; *SqCC* = squamous cell carcinoma; *H&N* = head and neck; *fx* = fractions; *A* = alive; *D* = dead.

### Dosimetric analysis

**Figure 5:**
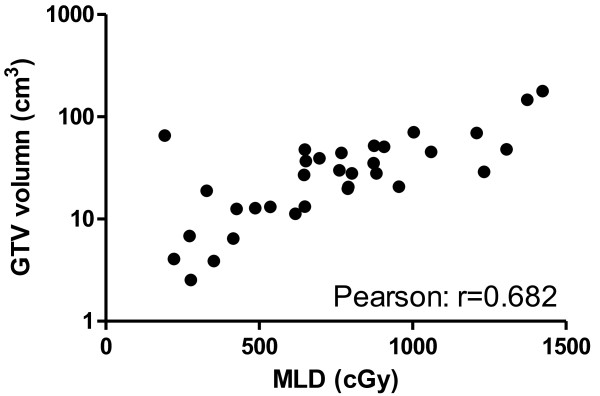
MLD vs GTV.

**Figure 6:**
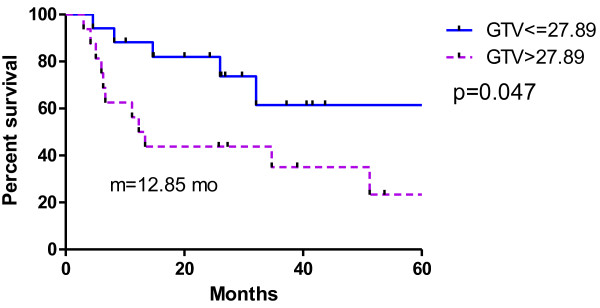
Use GTV volume to predict survival.

The median GTV was 27.89 cm^3^ (range: 2.54–178.08 cm^3^). There was a weak positive linear correlation between MLDs and GTVs, with a Pearson correlation of 0.682. This correlation is illustrated in Figure 5. Patients with a smaller sum of intrapulmonary GTVs seemed to have better OS, as shown in Figure 6. Those whose GTVs were smaller than the median value of 27.89 cm^3^ had a median survival > 40 months (not reached), whereas the patients with a GTV larger than 27.89 cm^3^ had a median survival of only 12.85 months (p = 0.047).

### Acute and late toxicity

**Figure 7:**
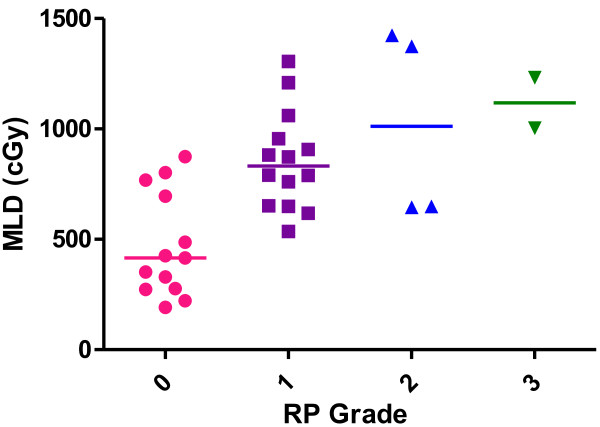
MLD vs RP.

During the follow-up examination, we found that 13 patients (39.4%) had grade 0 RP, 14 (42.4%) had grade 1 RP, and 4 (12.1%) had grade 2 RP. There were 2 patients (6.1%) who developed ≥ grade 3 RP. The median value of MLD in the grade 0 RP group was 415.0 cGy; in the grade 1 RP group, 831.5 cGy; in the grade 2 RP group, 1011.5 cGy; and in the grade 3 RP group, 1118.5 cGy. The RP grade increased from grade 0 to 3 with a corresponding increase in the median value of MLD. These data are illustrated in Figure 7.

## Discussion

This study reports our experience of hypofractionated Tomotherapy for the treatment of oligometastatic primary or secondary lung cancer. We found that HRT in combination with systemic treatment may be effective without causing major toxicities. EPD status strongly predicted survival. To date, few studies have investigated the use of hypofractionated Tomotherapy for advanced-stage primary lung cancer. Adkison et al. [13] reported the effectiveness of hypofractionated Tomotherapy for inoperable NSCLC with 2.28–3.22 Gy per fraction, for a total of 25 fractions in 46 patients. The median survival time was 18 months, with an actuarial 2-year survival of 46.8% and a 6.5% in-field progression rate. However, 80% of their patient population had stage III cancers. Song et al. [14] reported a 2-year survival rate of 56% and a 2-year LC rate of 63% in 37 NSCLC patients after hypofractionated Tomotherapy with 2.0–2.4 Gy per fraction, at a total dose of 60–70.4 Gy. Again, 75.7% of their patients had stage III cancer, 13.5% had stage I and II cancers, and 10.8% had recurrent disease. However, all of the patients in our study population had stage IV cancer and achieved a 2-year survival rate of 55.9% and a median survival of 32.1 months. We believe that one of the reasons for our results is that most of our lung cancer patients responded to the systemic targeted therapy.

Patients with oligometastatic lung tumors without extrapulmonary disease (EPD) had better survival than those with EPD, regardless of treatment modality. Zhang Ye et al. [15] reported that in patients with secondary lung cancers who underwent SBRT, the 5-year survival rate (33.2% vs 12.5%), median survival time (37.3 months vs 18.2 months, p = 0.012), and hazard ratio (HR, 1.894; p = 0.024) all favored negative EPD. Yamakado et al. [16] presented a study of colon cancer with lung metastasis treated with radio frequency ablation (RFA). They found that the 5-year survival rate for the patients without EPD was 57%, whereas that for the patients with EPD was 0% (p < 0.0001). Surgical management of oligometastases in esophageal cancer patients by Ichikawa et al. [17] also revealed that EPD was the most important prognostic factor. The 3-year survival rates were 54.7% for EPD-negative patients and 0% for EPD-positive patients (p = 0.0411). A large series of pulmonary metastasectomy for CRC conducted by Kanemitsu et al. [18] also demonstrated that EPD was a poor prognostic factor, with HRs of 1.73 (univariate, p = 0.001) and 1.55 (multivariate, p = 0.021). The study conducted by Schuhan et al. [19], which evaluated survival after pulmonary metastasectomy in malignant melanoma, disclosed that before surgery, EPD-negative patients had a median survival time of 39.8 months, compared with the 15.7 months (p = 0.23) in those with EPD. All of these studies generated the same conclusion as ours: that the median survival time of patients with EPD was 11.2 months, as compared with the >50 months (not reached) in patients without EPD (p < 0.001).

The importance of the involved numbers of organs or lesions in survival outcome was reported in 2 studies [20,21]. On the contrary, a pilot study for SBRT conducted by Milano et al. [21] disclosed that neither the numbers of organs nor lesions involved could predict survival (univariate, p = 0.43; multivariate, p = 0.50). Kim et al. [20] reported that treatment of oligometastatic lung tumors with Tomotherapy resulted in better overall survival in patients with ≤5 intrapulmonary lesions than those with >5 lesions, but the difference was not statistically significant (17 months vs 10 months, p = 0.2323). We observed a median OS time > 40 months versus 26 months in those with ≤2 versus those with ≥3 intrapulmonary lesions, respectively (p = 0.065).

Milano’s [21] prospective study also concluded that smaller GTVs were significant for LC, distant control, and survival benefit. Another long-term follow-up study by Milano [22] also showed that greater SBRT target volume in non-breast cancer was a poor prognostic factor for OS (p = 0.012). Our study also revealed that a small intrapulmonary GTV (≤27.89 cm^3^) correlated with better survival than a large GTV (≥27.89 cm^3^): >40 months versus 12.85 months (p = 0.047). These results are in accordance with the rationale of oligometastasis that higher tumor burden beyond the threshold size leads to an exponential rise in cell proliferation and the risk of distant metastasis. GTV has proved to be more significant than the number of metastatic lesions or organs for predicting survival.

Stage IV lung cancer is a systemic disease. The survival benefit from good LC should be based on good systemic control, and high LC rate alone would be meaningless in systemic disease. We have previously reported [23] that patients with mainly stage IV lung adenocarcinoma may gain longer survival by taking concurrent TKI plus multi-target Tomotherapy with a smaller fraction size, that is, 250 cGy/fx, for a total of 20–25 fractions. The 3-year overall survival rate reached 62.5%, and the progression-free survival (PFS) time was 16 months. The results of this study implied that early intervention of oligometastasis by radiotherapy in patients with fair systemic control may prolong progression-free survival and probably offer a greater survival benefit than systemic therapy alone. In the present study, we reviewed patients treated with Tomotherapy with a higher fraction size (450–700 cGy per fraction) and found a similar long survival rate but a more impressive LC rate.

The outcome of oligometastatic primary or secondary lung cancer treatment depends mainly on the nature of the tumor and the response to systemic treatment. Ideally, the oligometastatic state should be more indolent in nature. Metastases that are limited in number and location may be due to the fact that the metastatic machinery has not matured at that time. Disease progression is just a matter of time, and tumors detected in the oligometastatic state may be more vulnerable to therapy. An aggressive approach such as early intervention by local or multi-target radiotherapy in addition to mainstream systemic therapy is important to avoid malignant progression and drug resistance in response to systemic therapy alone.

In our study, the RP grade was quite low, with only 2 patients (6.1%) having ≥3 RP and most patients having grade 0 or 1 RP (81.8%), which is comparable with other studies. Park et al. [24] evaluated the early CT findings in 25 patients with pulmonary malignancies treated by Tomotherapy. The median total dose was 50 ± 4.99 Gy in 3–20 fractions. They reported that none of the patients developed ≥ grade 3 RP. Adkison et al. [13] treated 46 lung cancer patients with hypofractionated Tomotherapy with 2.28–3.22 Gy per fraction, for a total of 25 fractions. They also reported a ≥ grade 3 RP rate of 0%. Song et al. [14] described a trial in which 37 NSCLC patients treated with Tomotherapy with 2.0-2.4 Gy per fraction, up to total 60–70.4 Gy, exhibited a higher ≥ grade 3 RP rate (19%). Generally, SBRT of smaller GTV yields a lower ≥ grade 3 RP rate of 1.2–4% [25,26]. Referring to the report from Vofelius et al., Tomotherapy is particularly safe in the range of 7 to 20 fractionation schedule (RP risk ≦8%), while RP rate by 3D-CRT technique would be 10-12% in 7 to 20 fractions and even up to 13-16% in ≦5 fractions [27].

The median fraction size of 4.5–6 Gy/fx to a median total dose of 49.5 Gy was performed in our study. We had a lower BED (median estimated BED_10_ was 71.78 Gy) than that provided by the widely used high-fraction size SBRT technique (median BED_10_ ≥ 100 Gy in most studies). Nonetheless, a 94.7% 2-year LC rate and a 6.1% severe RP rate make our protocol quite attractive. Tomotherapy with conventional immobilization casts is much more convenient than SBRT with a stereotactic body frame. Furthermore, hypofractionated Tomotherapy can treat a larger tumor size. It can also treat multitargets in the thorax and extrapulmonary regions at the same time.

## Conclusion

The heterogeneity of tumor, small number of patients and retrospective data collection are the 3 important limitations of this study. Nevertheless, we can conclude a few important observations. Firstly, hypofractionated Tomotherapy with conventional immobilization cast may be as good as SBRT with extreme hypofractionation. Secondly, oligometastasis confined in lung organ only has better result than more than 2 organs involvement. GTV volume is more significant than number of metastatic lesions or organs involvement in predicting survival. Thirdly, oligometastatic state created from era of successful systemic treatment may highlight the importance of early integrating HRT with systemic treatment in primary and secondary lung cancer patients.

## Abbreviations

BED: Biological effective dose; GTV: Gross tumor volume; MLD: Mean lung dose; EPD: Extra-pulmonary disease; RP: Radiation pneumonitis; TKI: Tyrosine kinase inhibitor; SRT: Stereotactic radiation therapy; SBRT: Stereotactic body radiation therapy; HRT: Hypofractionated radiotherapy; NSCLC: Non-small cell lung cancer.

## Competing interests

None of the authors report any competing interests.

## Authors’ contributions

KHC is responsible for the study design. CHJ, HLK, CYL, SJK, YWY collected the data and contributed the cases. CHJ created the manuscript draft and RHW supervised the medical physics. All authors read and approved the final manuscript.
